# Cross-platform certification of the qubit space with a minimal number of parameters

**DOI:** 10.1038/s41598-025-27248-7

**Published:** 2025-12-05

**Authors:** Tomasz Rybotycki, Tomasz Białecki, Josep Batle, Jakub Tworzydło, Adam Bednorz

**Affiliations:** 1 Systems Research Institute, Polish Academy of Sciences, 6 Newelska Street, PL01-447 Warsaw, Poland; 2 Nicolaus Copernicus Astronomical Center, Polish Academy of Sciences, 18 Bartycka Street, PL00-716 Warsaw, Poland; 3 Center of Excellence in Artificial Intelligence, AGH University, 30 Mickiewicza Lane, PL30-059 Cracow, Poland; 4 Faculty of Physics, University of Warsaw, ul. Pasteura 5, PL02-093 Warsaw, Poland; 5 Departament de Física, Institut d’Aplicacions Computacionals de Codi Comunitari (IAC3), Campus UIB, E-07122 Palma de Mallorca, Balearic Islands Spain; 6 CRISP – Centre de Recerca Independent de sa Pobla, 07420 sa Pobla, Balearic Islands Spain

**Keywords:** Qubits, Quantum mechanics

## Abstract

We demonstrate a determinant dimension witness of a qubit space. Our test has a minimal number of independent parameters. We achieve it by mapping the Bloch sphere $\pi /2$-rotation axis angle on the non-planar so-called Viviani curve. We ran our test on different platforms: IBM Quantum, IQM Resonance, and IonQ. Our investigations show that numerous qubits, especially from the newest IBM Heron family devices, fail the test by more than ten standard deviations. The nature of those deviations has no simple explanation as the test is robust against common imperfections.

## Introduction

Quantum computers rely on two-level systems, commonly known as qubits. Computations require the qubit space to be reliable, not combined with a larger space in particular. The limit on the computational space dimension is critical for a fault-tolerant quantum computation performed on real devices, as the error correction relies on the restricted model of noise^1–3^.

Quantum space dimension can be verified by a dimension witness^4–9^. Such witnesses implement a two-stage protocol – the initial preparation and the final measurement, both chosen independently. A special case of dimension witnesses are null witnesses^10^. For a system below a specified dimension, the value of such witness is – up to statistical error – zero, and nonzero otherwise. In a sense, a null witness certifies the linear independence of the outcome probability *p*(*M*|*P*) for the preparation *P* and measurement *M*^10–13^.

Dimensionality tests are robust against several errors of existing quantum gates. Microwave pulses, used to perform physical operations on the qubits, suffer from nonlinearities of waveform generators^14^. Therefore, a simple deviation of the probability distribution from the theoretical prediction^15^ is not a sufficient certification of an extra qubit space. We have already performed such tests on IBM Quantum^16^, and its variant for a single repeated gate^17^, and found significant deviations.

In the present contribution, we simplify the witness further^11^, with just two independent parameters (angles) to control the preparation and measurement. Such approach minimizes the possible correlations between the preparation and measurement parameters. We tested a set of qubits at various times, on multiple platforms: IBM Quantum, IonQ, and IQM Resonance. The main advantages of this test are:

- high precision,
- minimal preparation-measurement correlation,
- no two-qubit gates,
- robustness against relaxation and dephasing errors,
- cross-platform compatibility,
- reproducibility.

Some systems passed the experimental tests, and for them the two-level qubit model holds. However, there are many cases failing the test at 5–10 and more standard deviations, the newest IBM Heron computers in particular. Although test results vary in time, for IBM Heron family devices the deviations are generally consistent. Some qubits tested a month later passed the test, while the others failed. Moreover, the deviations on IonQ devices occur incidentally. This result shows that one cannot detect the cause in the routine calibration. Whatever the reason, it needs an urgent explanation to advance efforts of efficient error correction.

## Description of the test

The test is applied to the qubit space $d=2$ with the witness constructed from the measurement probabilities for the state *P* and the measurement operator *M*, associated with one of the outcomes, say 0. According to quantum theory, the probability reads $p(M|P)=\textrm{tr}M P$, where the preparation operator is $P=P^{\dag }\ge 0$, $\textrm{tr}P=1$ and the measurement is $1\ge M=M^{\dag }\ge 0$.

We propose a determinant dimension witness test based on a $5 \times 5$ matrix of probabilities *F*. The entries of *F* are $F_{kj}=p(M_{j}|P_{k})$ for $k = 1, 2,\ldots , 5$ and $j = 1, 2, 3, 4.$ Since we need 5 parameter values for state preparation but only 4 values for the measurement, we need to supplement the final row with ones, formally $F_{5j}=1$. This way we still expect determinant witness $W := \det F$ to be 0 in the ideal case, that is if all $P_{j}$ and $M_{k}$ represent the same two-level space^11,18^. This is a straightforward consequence linearity of the trace with respect to the matrices, which have maximally $d^2$ entries in the quantum space of *d* dimensions. Exceeding this limit makes columns/row linearly dependent and the determinant must vanish.

Moreover, the determinant remains zero even if all preparations contain some constant incoherent leakage term, namely $P'_{j}=P_{j}+{\tilde{P}}$, with ${\tilde{P}}$ in an arbitrary space, independent of *j*. In this sense, the uncontrolled leakage to an extra space does not affect the test^18,19^.

If the quantum system consists of only two levels (like qubit), we expect $W=0$. Any deviation should remain within the statistical error due to finite statistics. Otherwise, the deviation would indicate that the actual space is larger. Assuming a two-dimensional Hilbert space, we construct the states and the operators in the computational basis $|0\rangle$, $|1\rangle$. We also assume the initial state is $|0\rangle \!\langle 0|$. Additionally, the test relies on the following assumptions:

- independent and identical probability distributions of all repetitions,
- independence between the preparation and measurement operations.

Since we aimed to run our test on a real devices, we had to decide what gates to use in our test. It would benefit the experiment greatly, if the gates were native to the target device(s), since then the gate-induced error would be the lowest. Previously, we tested IBM Quantum devices^15–17^, so we had the most experience with them. We decided to use the devices’ native gates, i.e. use the simplest steering pulse sequences when constructing the quantum circuits, not only for IBM but also IQM and IonQ. The native gate realized by IBM Quantum, IQM and IonQ is the $\pi /2$ rotation on the Bloch sphere in $\{|0\rangle$, $|1\rangle \}$ space:

1 $$\begin{aligned} S=\frac{1}{\sqrt{2}} \begin{pmatrix} 1 & -i \\ -i & 1 \end{pmatrix}. \end{aligned}$$

Moreover, the rotation axis can itself be rotated by a given angle $\theta$. This rotation is realized by an *S* gate and two auxiliary $Z(\theta )$ (phase shift) gates,

2 $$\begin{aligned} S_{\theta }=Z^{\dag }_{\theta } SZ_{\theta } ,\, Z_{\theta }= \begin{pmatrix} e^{-i\theta /2} & 0 \\ 0 & e^{i\theta /2} \end{pmatrix}. \end{aligned}$$

**Fig. 1:**
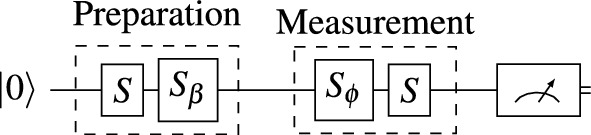
The quantum circuit for the dimension test with two parameters, angles $\beta$ and $\phi$. The protocol starts from the initial state $|0\rangle$, followed by preparation phase, gates *S* and $S_{\beta }$ and the measurement phase, $S_{\phi }$ and *S*, with the final readout in the computational basis.

The physical experiment describing our test is a sequence of qubit state $|0\rangle$ initialization, followed by *S*, $S_{\beta }$ gates (preparation), then the two gates $S_{\phi }$, *S* (measurement), and the readout pulse for the measurement of the state $|0\rangle$ again, see Fig. 1. The parameters are 5 preparation angles $\beta _{j}$ ($j=1..5$) to be chosen independently of the 4 measurement angles $\phi _{k}$ ($k=1..4$). It gives the prepared state $P_{\beta }=S_{\beta } S|0\rangle \!\langle 0|S^{\dag } S^{\dag }_{\beta }$ and the measured state $M_{ \phi }=S^{\dag }_{\phi } S^{\dag }|0\rangle \!\langle 0|S S_{\phi }$, in terms of operators. We also have to assume that the gates/operations do not depend on the previous ones. To sum up, we can only test the combination of assumptions: dimension of the space and independence of operations.

One can use Bloch sphere representation of prepared states and measurements, using vectors ${\varvec{n}}$ to represent the state $P=|{\varvec{p}}\rangle \!\langle {\varvec{p}}|=(1+{\varvec{p}}\cdot \varvec{\sigma })/2$, with Pauli matrices $\sigma _{1,2,3}$. The initial state $|0\rangle \!\langle 0|$ corresponds to the vector (0, 0, 1). The $\pi /2$ rotations by *S* gate and phase shifts $Z_{\theta }$ can be used to obtain the non-planar parametric Viviani curve on the Bloch sphere. Let us recall that the Viviani curve arises as the intersection of a cylinder tangent to a sphere and embedded inside it, passing through the origin. In our specific operation sequences, the vectors ${\varvec{p}}_{\beta }$, ${\varvec{m}}_{\phi }$, form respective opposite Viviani curves $-(\sin \beta \cos \beta ,\sin ^{2}\beta ,\cos \beta )$ and $(\sin \phi \cos \phi ,\sin ^{2}\phi ,\cos \phi )$. Viviani curves are especially useful in our test because:

- it is non-planar, meaning it spans complete qubit space, in contrast to simple circles,
- they are realized by a native gates with single parameter ($\beta$ and $\phi$) minimizing the unwanted correlations between the preparation and measurement operations,
- the actual sets of angles are chosen to make the adjugate matrix $\textrm{Adj}\,F$ (contrary to inverse matrix, the adjugate one exists even if $W=0$) distant from zero as possible to increase the test’s sensitivity to potential external states.

The restriction to Viviani curve is not obligatory. One is free to choose any set of preparations and measurements, similarly to^16^. However, our goal was to construct a simple cross-platform qubit dimension test, hence the choice of Viviani curve angles.

Then the probability matrix elements read

3 $$\begin{aligned} F_{kj}=\textrm{Tr}M_{k}P_{j}=(1+{\varvec{p}}_{j}\cdot {\varvec{m}}_{k})/2. \end{aligned}$$

**Fig. 2:**
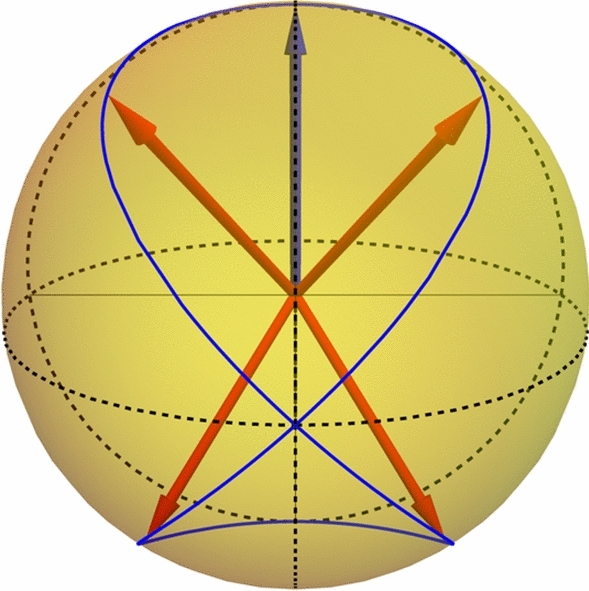
The Bloch vectors for the preparations ${\varvec{p}}$ (red and blue arrows) and measurements ${\varvec{m}}$ (red arrows) corresponding to the angles used in the test, positioned on the Viviani curve (blue curve).

We used

$$\beta =\{\pi /4, -\pi /4, 3\pi /4, -3\pi /4, 0\},$$

and $\phi _{k}=-\beta _{k}$ for $k=1\dots 4$. Respective Bloch vectors and their positions on the Viviani curve are presented in Fig. 2.

Theoretically, we can calculate the probability for every $\beta$ and $\phi$. Practically, the test is stochastic, and we have to estimate the error due to finite statistics. From Laplace expansion

4 $$\begin{aligned} \delta W=\sum _{kj}\delta F_{kj}(\textrm{Adj}\,F)_{jk} \end{aligned}$$

where Adj is the adjugate matrix, and $\delta F= F-\langle F\rangle$ is the deviation of empirically obtained probability (frequencies) matrix *F* from its theoretical form. The variance of *W* is

5 $$\begin{aligned} T\sigma ^2=T\langle W^2\rangle \simeq \sum _{kj}F_{kj}(1-F_{kj})(\textrm{Adj}\, F)_{jk}^2, \end{aligned}$$

where *T* is the experiment repetitions number^11^. The data we collected allow estimating the above error, which is necessary for the results confidence level estimation / determination.

**Fig. 3:**
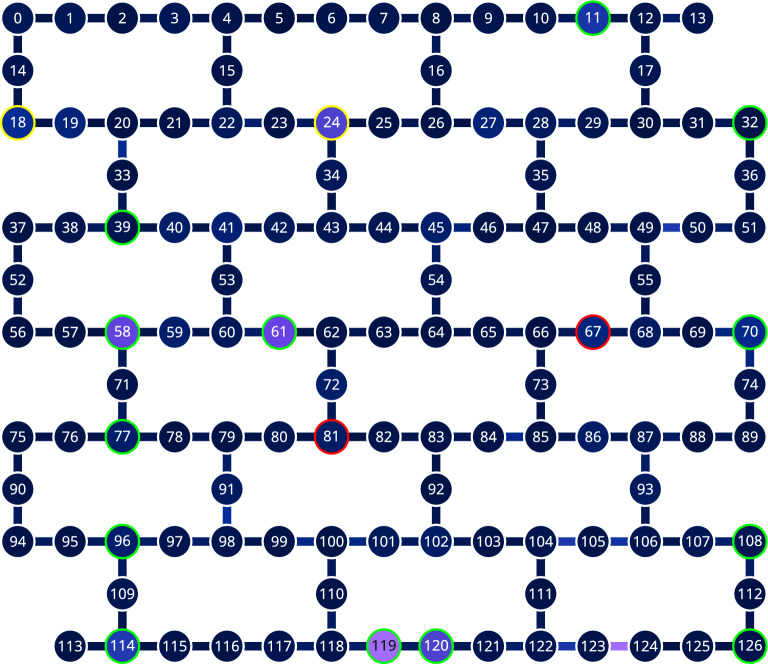
The topology of ibm_brisbane device. We highlighted the most faulty qubits tested in February – yellow, and in March 2024 – red. The rest of the qubits, highlighted green, have passed the test. Two-qubit Echoed Cross Resonance gates, unused in the test, connect the qubits.

**Fig. 4:**
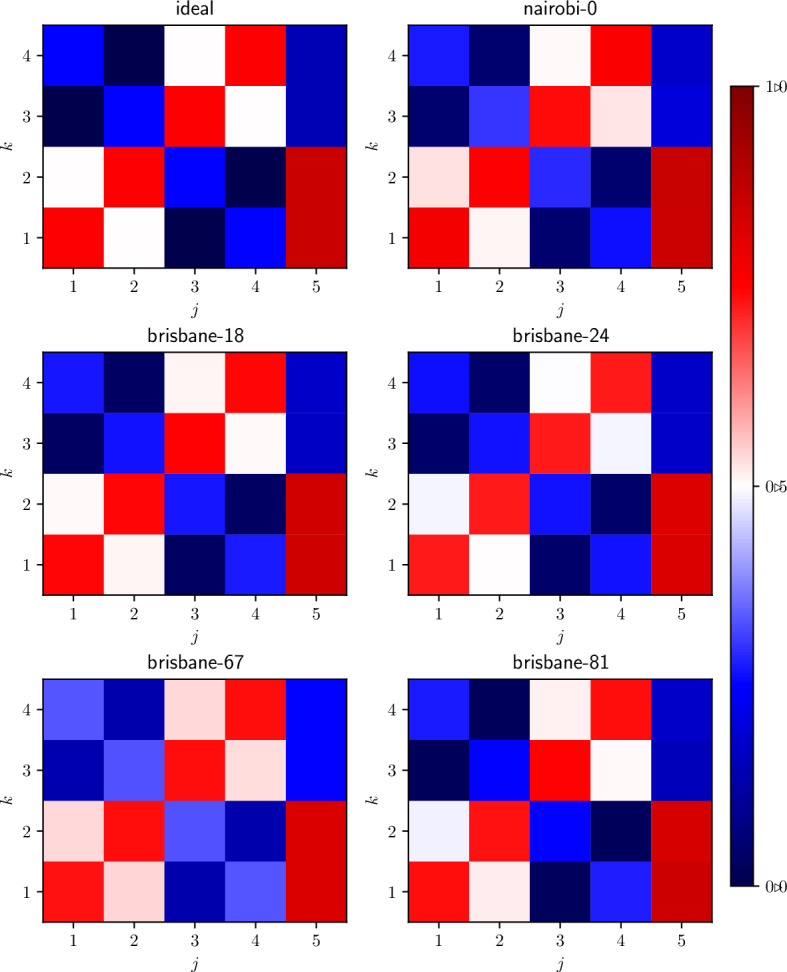
The probabilities $F_{kj}$ for the test using selected quantum states and measurement represented by Bloch vectors on the Viviani curve. From the top-left: ideal, ibm_nairobi qubit 0, ibm_brisbane qubits 18, 24 (Feb 2024), and 67, 81 (Mar 2024).

**Fig. 5:**
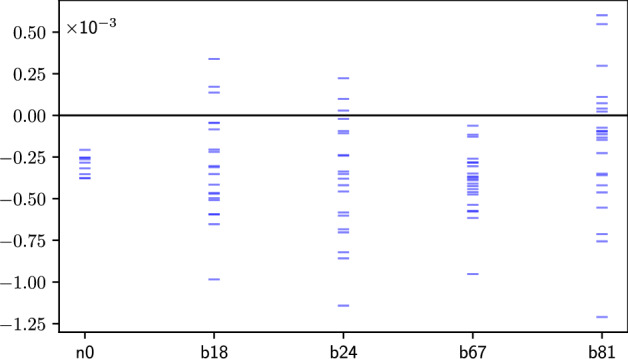
The values of the witness for individual jobs: ibm_nairobi qubit 0, ibm_brisbane qubits 18, 24 (Feb 2024) and 67, 81 (Mar 2024). We use symbols $n\#$ and $b\#$ for ibm_nairobi and ibm_brisbane qubits respectively.

## Experiments

We have run the above test on three publicly available quantum computing platforms: IBM, IQM, and IonQ. We used identical circuits and randomly shuffled the angles. Our test doesn’t require a perfect implementation of gates. Standard local sources of depolarizing and relaxation errors, and general gate and readout errors do not affect our test, as they are assumed to remain within the two-level space. Moreover, the test accounts for leakage to external states (e.g., $|2\rangle$) as long as it is incoherent and does not depend on the circuit parameters $\beta ,\phi$. That is because occurrence of such phenomenon adds a global constant to each $F_{jk}$^18,19^. We present the comparison of single qubit gate properties for respective platforms in Table 1.

Due to nonlinearity of determinant and to avoid calibration dependence, we have calculated *W* using data from IBM and IQM in two ways:

(i) determining *F* for each job (basic complete computation task unit), finding determinant $W_{\text {job}}$ for each job, and finally averaging over $W_{\text {job}}$,
(ii) first averaging *F* over all jobs and then computing *W*.

The latter approach failed on IonQ because of large drifts in the data. We suspect varying calibrations were the cause of the drift.

**Table 1:** Comparison of qubit and gate properties for different platforms: single-qubit rotation gate time, qubit measurement time, relaxation decay time T1, decoherence time T2, single qubit gate error. The IBM families, Falcon (ibm_nairobi), Eagle (ibm_brisbane), Heron (ibm_torino, ibm_pittsburgh) differ rather by architecture and overall performance, while the single-qubit operations change within the similar order of magnitude.

platform	gate [s]	measure [s]	T1 [s]	T2 [s]	gate error
IBM	$5\cdot 10^{-8}$	$1\cdot 10^{-6}$	$\sim 10^{-4}$	$\sim 10^{-4}$	$\sim 10^{-4}$
IQM	-	-	$\sim 10^{-5}$	$\sim 10^{-5}$	$\sim 10^{-3}$
IonQ	$\sim 10^{-4}$	$5\cdot 10^{-5}$	10	1.5	$5\cdot 10^{-4}$

**Fig. 6:**
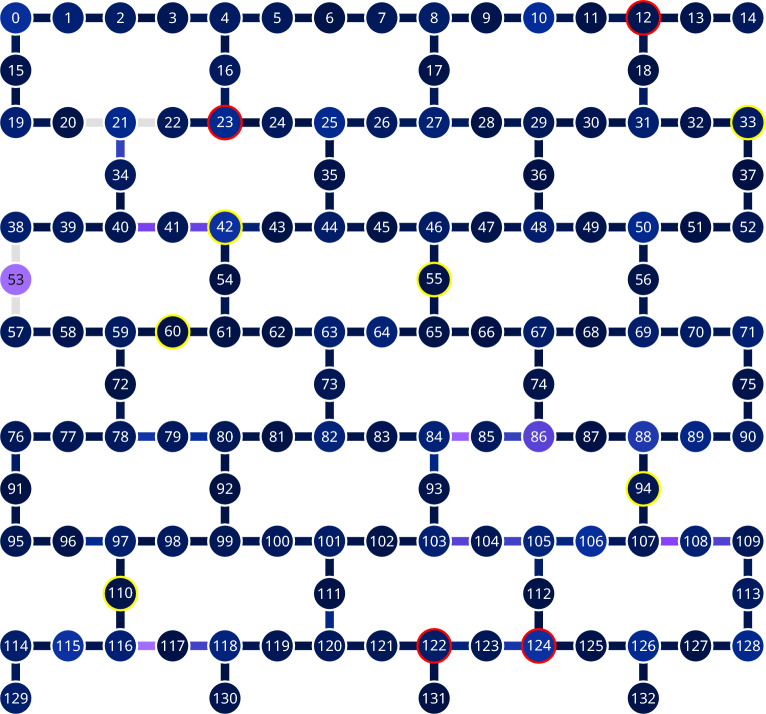
The topology of ibm_torino device. We highlighted the qubits tested in September 2025 with yellow and red. The latter show exceptionally large violation.

**Fig. 7:**
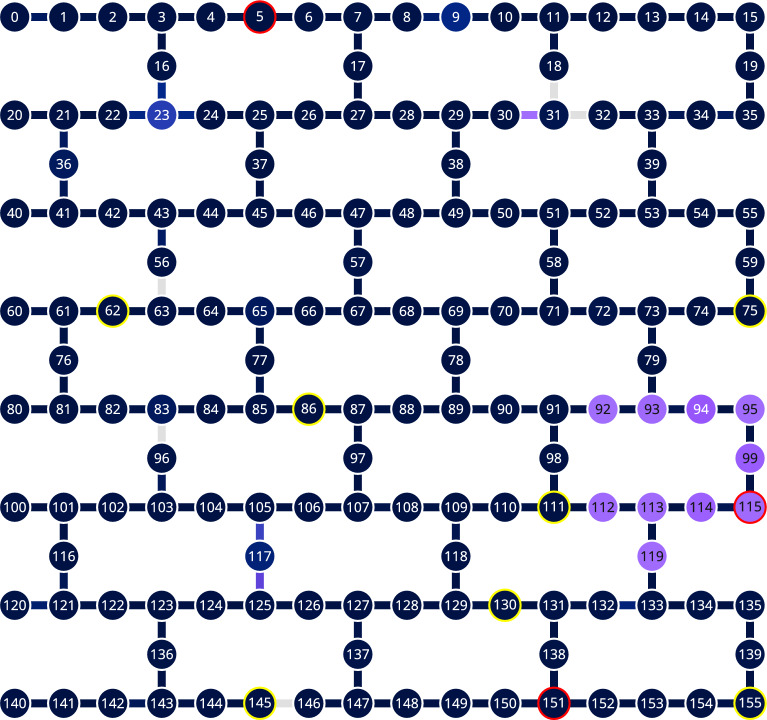
The topology of ibm_pittsburgh device. We highlighted the qubits tested in September 2025 with yellow and red. The latter show exceptionally large violation.

The data and scripts used in the experiments are available at the public repository^31^.

### Hardware specification

The IBM Quantum, IonQ, and IQM Resonance platforms offer a list of devices consisting of many qubits. The qubits are controlled by a user-defined set of gates (operations) – either single-qubit or two-qubit ones, parametrized by phase shifts.

The IBM and IQM qubits are physical transmons^20,22^, i.e. artificial quantum states existing due to the interplay of superconductivity (Josephson effect) and capacitance (Coulomb charging effect). The transmon consists in principle of a sequence of levels $|0\rangle$, $|1\rangle$, $|2\rangle$, and so on, denoted shortly as $0,1,2,\dots$. For (contemporary) quantum computers to work correctly, we expect the devices to restrict their operations range to $|0\rangle$ and $|1\rangle$. On the other hand, the IonQ qubits are trapped ytterbium ions in the space spanned by two hyperfine-split states^21^.

Each gate is realized by a microwave pulse of the drive frequency (equivalent to energy difference between $0-1$ levels) for transmons or alternatively by Raman transitions for ions,^23^. The pulse frequency for transmons is in the range $4-5$ GHz, and $\sim 12$GHz for ions. It is worth mentioning, that the $Z_{\gamma }$ gate is not actually a real pulse, but is realized by adding a rotation (phase shift) between real and imaginary components of the pulse for upcoming gates^24^. In the case of transmons, due to the anharmonicity (difference between $0-1$ and $1-2$ transition frequencies) of about 300MHz, the working space effectively collapses into two states 0 and 1.

The readout of transmons is performed by coupling the resonator to another long microwave pulse at a frequency different from the drive frequency, and finally measuring the populated photons^23,25^. Ions are measured by laser pulse stimulating transitions to specific levels.

### IBM simulations

To ensure our test works as intended, we ran it on the qiskit aer simulator. First we assumed the perfect gates and no noise. This test was done on a single qubit, because without the noise, simulator qubits are indistinguishable. We ran a single job with $10^6$ shots. We got $W_{\textrm{sim}} = (-13.8 \pm 9.9) \cdot 10^{-5}$.

**Fig. 8:**
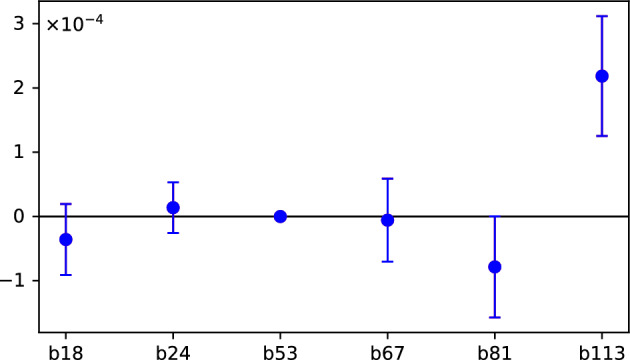
The value of the witness *W* of the selected simulated ibm_brisbane qubits denoted b*n*, with *n* being the qubit number. Aside from the qubits we used for the experiment on a real device, we also present the least (b53) and the most (b113) noisy qubit.

We then repeated our tests, this time on a noisy simulator. We targeted each qubit of the ibm_brisbane backend. Since the gate errors vary from qubit to qubit, we wanted to see if this variation will show up in the simulations. We ran a single job with $10^6$ shots for each qubit. The results are shown in the Fig. 8.

We can see that even on the simulator the disproportion between qubits performance is significant. While the least noisy qubit had the witness value of order $10^{-8}$, the value of *W* for the most noisy one was of order $10^{-4}$. We have rerun the simulations in September 2025 imitating IBM Heron devices. In the latest simulations, no siginificant deviations have been found.

### IBM

Initially, we ran the test on two IBM Quantum devices: ibm_nairobi and ibm_brisbane^28^. The first was a 7-qubit system on which we tested the qubit 0 in October 2023. The latter is a 127-qubit system. The declared coherence times are of the order $50\cdot 10^{-6}$ s, while the single-qubit gates take $\sim 50\cdot 10^{-9}$ s and readout $\sim 1.2\cdot 10^{-3}$ s. To use it efficiently, we have chosen two sets of 10 qubits, the first in February and the second in March 2024.

To run the experiments on IBM devices, one sends a list of so-called jobs for execution. Experiments can consist of multiple jobs, which can be repeated a number of times. Aside from defining quantum circuits to be executed on the target device, one has to specify additional job parameter. One of those, the number of shots, specifies the repetition number of the preprogrammed sequence of circuits within a single job. Note that each execution of a shot means running full sequence of circuits, then proceeding to the next shot. To avoid memory-related effects, we randomly shuffled the experiments within each job. The total number of each circuit executions is thus given by $T=\#\textrm{jobs}\cdot \#\textrm{shots}\cdot \#\textrm{repetitions}$. The readout count for each circuit is the value returned after the job execution is complete.

For ibm_nairobi, we ran nine jobs at $10^5$ shots and 15 repetitions. For ibm_brisbane, we ran 20 jobs in February and 22 in March 2024, with $10^4$ shots and ten repetitions each, because of access limitations enforced by IBM. We could measure ten qubits simultaneously, treating them as independent, because their positions on the qubits grid are isolated, see Fig. 3. Moreover, the random shuffling of the parameters was applied to each qubit independently, so any crosstalk is unlikely.

In Tables 3 and 2 , we present the results and parameters of the qubits for both runs. The tests on ibm_nairobi qubit 0 and ibm_brisbane qubits – 18, 24 in February and 67, 81 in March 2024 – resulted in the witness’s value at five or more standard deviations. These cases are presented in detail in Fig. 4 (the matrix of probabilities) and in Fig. 5 (results of individual jobs).

As of late, introduced newer, Heron family devices: ibm_torino with 133 qubits and ibm_pittsburgh with 156 qubits. In September 2025, we have run analogous tests on both of them, with 40 and 30 jobs, respectively. We chose the qubits shown in Figs. 6 and 7 . The tests showed significant violations with negative determinant *W* values, see Table 4.

We stress that the gate error of order $10^{-4}$ could not cause such deviations. That is because in such case the deviations should be of the order of the square of the gate errors $\sim 10^{-8}$ (adding simultaneously to the preparations and measurements). The temporal change in the witness value hints that the cause comes from the device calibrations, not just the pulse frequencies, which vary only slightly. However, consistent and large negative-valued violation of the dimension witness in Heron devices indicate a serious operational issue that has to be investigated. The leakage to higher states, i.e., $|2\rangle$, is negligible^15,18^, as its effects are much below the gate errors (see also our discussion in^16^).

**Table 2:** Experiment results for ibm_nairobi qubit 0 (test in October 2023). The data contain drive frequency (inter-level), error of the *S* gate, the witness $W^{i/ii}$ and the standard deviation $\sigma ^{i/ii}$. Indices *i* and *ii* of *W* and $\sigma$ indicate the *W* calculation strategy discussed in section . Faulty qubits, with *W* nonzero beyond 5 standard deviations, are bolded.

drive freq. (GHz)	gate error $10^{-4}$	$W^{i}[10^{-5}]$	$\sigma ^{i}[10^{-5}]$	$W^{ii}[10^{-5}]$	$\sigma ^{ii}[10^{-5}]$
5.26	2.5	**-29.8**	**2.3**	**-29.9**	**2.3**

**Table 3:** Experiment results for respective ibm_brisbane qubits (tests in February and March 2024). The data contain drive frequency (inter-level), error of the *S* gate, the witness $W^{i/ii}$ and the standard deviation $\sigma ^{i/ii}$. Indices *i* and *ii* of *W* and $\sigma$ indicate the *W* calculation strategy discussed in section . Faulty qubits, with *W* nonzero beyond 5 standard deviations, are bolded.

qubit (February 2024)	**18**	**24**	32	39	58	61	70	114	119	126
drive freq. [GHz]	4.788	5.101	4.910	4.917	4.887	4.794	4.899	4.822	4.803	4.908
gate error $[10^{-4}]$	2.1	1.6	2.9	2.0	2.2	6.2	2.9	2.0	3.2	2.4
$W^{i}[10^{-5}]$	**-30**	**-39**	-18	-1.2	1.7	-3.6	-18	-1.2	-17	19
$\sigma ^{i}[10^{-5}]$	**6.1**	**5.5**	6.4	5.4	6.5	6.2	4.5	2.6	5.2	6.5
$W^{ii}[10^{-5}]$	**-30**	**-38**	-18	-1.4	1.7	-3.6	-18	-1.3	-17	19
$\sigma ^{ii}[10^{-5}]$	**6.1**	**5.5**	6.4	5.4	6.5	6.2	4.5	2.6	5.2	6.5
qubit (March 2024)	11	18	24	**67**	70	77	**81**	96	108	120
drive freq. [GHz]	4.972	4.788	5.101	5.113	4.899	5.042	4.930	4.748	5.056	4.837
gate error $[10^{-4}]$	5.1	1.5	1.6	2.9	5.6	1.5	2.7	3.1	3.7	2.4
$W^{i}[10^{-5}]$	-9.6	-0.8	-16	**-40**	-19	-22	**-18**	-20	-3.2	-1.0
$\sigma ^{i}[10^{-5}]$	5.2	5.9	4.9	**3.8**	4.1	5.8	**2.6**	5.1	4.0	4.5
$W^{ii}[10^{-5}]$	-9.8	-0.9	-15	**-40**	-19	-22	**-18**	-20	-3.0	-1.0
$\sigma ^{ii}[10^{-5}]$	5.2	5.9	4.9	**3.8**	4.1	5.8	**2.6**	5.2	4.0	4.5

**Table 4:** Experiment results for respective IBM Heron devices, ibm_torino and ibm_pittsburgh qubits (test in September 2025). The data contain drive error of the *S* gate, the witness $W^{i/ii}$ and the standard deviation $\sigma ^{i/ii}$. Indices *i* and *ii* of *W* and $\sigma$ indicate the *W* calculation strategy discussed in section . Faulty qubits, with *W* beyond 20 standard deviations, are bolded. Drive frequencies for Heron family devices are not disclosed by IBM.

ibm_torino qubit	**12**	**23**	33	42	55	60	94	110	**122**	**124**
gate error $[10^{-4}]$	1.6	1.4	1.5	1.7	2.0	2.0	1.8	1.2	1.3	1.4
$W^{i}[10^{-5}]$	**-166**	**-288**	-50	-95	-80	-19	-22	-8.6	**-197**	**-233**
$\sigma ^{i}[10^{-5}]$	4	3.4	3.6	2.4	4.5	4.5	4.3	4.5	2.9	2.4
$W^{ii}[10^{-5}]$	**-158**	**-276**	-50	-91	-80	-19	-22	-8.6	-194	**-217**
$\sigma ^{ii}[10^{-5}]$	4	3.5	3.6	2.4	4.5	4.5	4.3	4.5	2.9	2.5
ibm_pittsburgh qubit	**5**	62	75	86	111	**115**	130	145	**151**	155
gate error $[10^{-4}]$	1.1	0.8	3.0	1.0	1.1	1.0	0.8	1.0	0.9	0.9
$W^{i}[10^{-5}]$	**-166**	-39	-52	-41	-72	**-110**	-52	-8.2	**-152**	-25
$\sigma ^{i}[10^{-5}]$	5.5	5.5	5.5	5.5	5.5	5.5	5.4	5.6	5.4	5.4
$W^{ii}[10^{-5}]$	**-165**	-39	-52	-40	-73	**-110**	-52	-8.1	**-152**	-25
$\sigma ^{ii}[10^{-5}]$	5.5	5.5	5.5	5.5	5.5	5.5	5.4	5.6	5.4	5.4

**Table 5:** Experiment results for respective IQM Resonance Garnet qubits (test in November 2024). The data contain error of the *S* gate, the witness $W^{i/ii}$ and the standard deviation $\sigma ^{i/ii}$. Indices *i* and *ii* of *W* and $\sigma$ indicate the *W* calculation strategy discussed in section . Faulty qubits, with *W* nonzero beyond 5 standard deviations, are bolded. Drive frequencies for IQM Resonance Garnet device are not disclosed by IQM.

qubit (November 2024)	1	**3**	**5**	9	13	**7**	**11**	**15**	18	17	**20**
gate error $[10^{-3}]$	1.1	0.9	1.6	1.2	1.0	1.3	3.0	1.3	0.6	1.6	0.6
$W^{i} [10^{-4}]$	-2.9	**-24.7**	**24.6**	-4.0	-0.4	**-6.3**	**-7.5**	**16.9**	-4.7	-3.0	**-17.2**
$\sigma ^{i} [10^{-4}]$	1.1	1.0	1.1	1.1	1.1	1.1	0.9	1.0	1.1	1.1	1.1
$W^{ii}[10^{-4}]$	-3.0	**-24.7**	**24.6**	-4.0	-0.4	**-6.2**	**-7.4**	**16.9**	-4.7	-3.1	**-17.2**
$\sigma ^{ii}[10^{-4}]$	1.1	1.1	1.1	1.1	1.1	1.1	0.9	1.0	1.1	1.1	1.1

### IQM

**Fig. 9:**
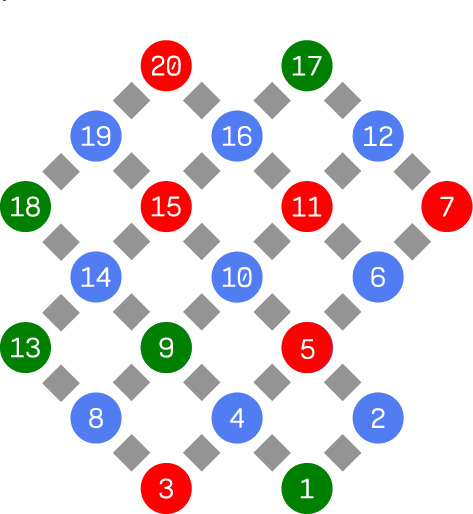
The topology of IQM Resonance Garnet. We highlighted the most faulty qubits tested in November 2024 with red. The rest of the qubits, highlighted with green, passed the test. Two-qubit CZ gates, unused in the test, connect the qubits.

In November 2024, we ran the same test on IQM Resonance - Garnet^29^, which contains 20 transmon qubits. The physical properties of this device are similar those of IBM devices. We have chosen 11 qubits so that none is next to the other, see Fig. 9. In this case we have run 13 jobs with $10^4$ shots and 5 repetitions.

The results turned out to be even worse than for IBM Quantum systems, with a half of the tested qubits faulty. The results are shown in Table 5. The largest value of the determinant witness *W* is $\sim 10^{-3}$, which is of the same order as single qubit gate error. Similarly to IBM Quantum, the simple leakage is an unlikely explanation.

**Fig. 10:**
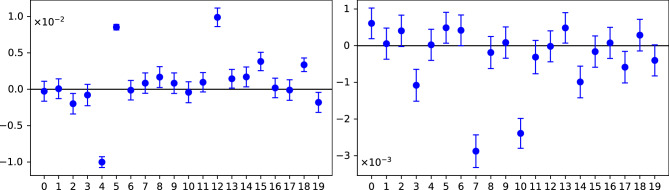
The values of the witness *W* of individual jobs on IonQ: aria-2 (top) and forte-enterprise-1 (bottom). Note that there are a few values differing from zero by more than 10 standard deviations.

### IonQ

The tests on IonQ aria-2 and forte-enterprise-1^30^ were a bit different. Due to larger single-qubit gate and readout times ($135 \cdot 10^{-6}$ s and $50 \cdot 10^{-6}$ s, respectively), but also coherence times $>1$ s, the tests take much longer. For that reason, the results become more sensitive to drifts and calibration. We have tested a single qubit 0 (ions are coupled all-to-all and are essentially identical), with 20 jobs, each one containing 5 repetitions and $10^3$ shots (meaning roughly 10 times smaller statistics compared to experiments with superconducting devices).

A single job took more than 1 hour, which is $>6$ times longer compared to 9 minutes on IBM/IQM. The jobs have been executed over 6 days in May 2024 on aria-2 and over 9 days in May 2025 on forte-enterprise-1. The results differed significantly from the ones obtained in the experiments with transmon devices. The witness is nonzero beyond 10 standard deviations for a few jobs, indicating sensitivity to calibrations or drifts, see Fig. 10.

## Conclusions

A test of the quantum system dimension by parametric rotations reveals significant deviations in various qubits of IBM Quantum, IQM Resonance, and IonQ devices.

Results from IBM and IQM seem insensitive to calibration drift as both methods of witness value averaging, (i) and (ii), lead to similar results. On the other hand, IONQ shows isolated deviations, that indeed may be related to changes in calibration. This needs further investigations. The deviations cannot result from conventional sources like leakage to higher excited states of neighbor qubits. This is especially puzzling for ion qubits which do not have a natural state to leak to. Moreover, their occurrence in a particular period suggests some relation to the unspecified calibration change. Large negative deviations found on IBM Heron devices need critical investigation, as they appear consistently across qubits and devices.

Further tests are necessary to identify the deviations’ origin, to exclude some sophisticated technical causes and more exotic options, e.g. many worlds/copies theories^32,33^. We suggest:

(i) collecting massive statistics in a relatively short time, which we simply cannot afford,
(ii) a test on all qubits simultaneously, which is currently impossible due to the implementation of classical registers (there is a single multi-outcome counter instead of single qubit counters),
(iii) a test on qubits implemented on additional platforms (different quantum providers or technology),
(iv) the use of single-qubit devices (to rule out the cross-talks),
(v) applying preparation and measurement on different qubits, with a swap in the middle.

Current results confirm that our previously found deviations^16^ were not just a fluke and deserve urgent explanation.

## Data Availability

The data are publicly available at doi.org/10.5281/zenodo.17223687.
